# Supplementary material for: Analysis of PPARγ Signaling Activity in Psoriasis
Source: Int J Mol Sci. 2021 Aug 10;22(16):8603. doi: 10.3390/ijms22168603 (PMC8395241; doi:10.3390/ijms22168603)
Supplement: Supplementary file 1 [file ijms-22-08603-s001.zip › Supplemental materials_Analysis of PPARg signaling activity in psoriasis/Pathway models/Models images and html files/Model of PPARG related pathways in psoriasis (short version)/9263.html]

AMPK


# Functional Class AMPK

|  |  |
| --- | --- |
| URN | urn:agi-enz:2.7.11.31 |
| Total Entities | 2 |
| Connectivity | 7751 |
| Name | AMPK |
| Description | AMP-dependent protein kinase |
| ObjectType | Functional Class |
| Mol Function | Protein kinases |

---

|  |  |
| --- | --- |
| ChildConcepts | PRKAA1 |
|  | PRKAA2 |

---

|  |  |
| --- | --- |
| Pathway | Longevity Related Drugs |
|  | Estrogen Deficiency in Female Obesity |
|  | Apoptosis of Cochlear Hair Cells (Noise-Induced, Mouse Model) |
|  | MTHFR Activity Regulation |
|  | DNA Persistent Repair Inhibits mTOR Signaling |
|  | mTOR Signaling Activation by Fatty Acids and Glucose |
|  | mTOR Signaling |
|  | mTOR Signaling Hyperfunction |
|  | Hypoxia Inhibits mTOR Signaling |
|  | SIRT1 Signaling in Aging |
|  | Autophagic-Lysosomal System Decline Associated with Aging |
|  | mTOR Signaling Activation by Taste Sensor Receptors (Rodent Model) |
|  | AMPK Related Catabolism Deceleration in Glucose Insufficiency |
|  | MTOR/TP53 Regulates Cell Metabolism |
|  | Insulin Influence on Protein Synthesis |
|  | Insulin Signaling |
|  | Vascular Endothelial Cell Activation by NO |
|  | BMP/TGF-beta Signaling Impairment in Pulmonary Hypertension |
|  | Adiponectin in Insulin Resistance Prevention |
|  | Insulin Resistance in Myocytes Induced by Obesity |
|  | Insulin Resistance in Hepatocytes |
|  | Nitric Oxide Effects on beta-Cell |
|  | IL6 and Insulin Resistance in Muscular Dystrophies |
|  | Ketogenic Diet Mechanism (Hypothesis) |
|  | Podocyte Dysfunction in Glomerulonephritis |
|  | PI3K/AKT/MTOR Signaling Activation by Blocking of Tumor Suppressors |
|  | Respiratory Chain Impairment in Cancer |
|  | Lactate as a Signaling Molecule in Cancer Cells |
|  | Metabolic Effects of Oncogenes and Tumor Suppressor in Cancer Cells |
|  | Lysophosphatidic Acid/LPARs Signaling |
|  | AMPK Signaling |
|  | mTOR Signaling |
|  | Neighbors of AMPK |
|  | Model of PPARG signaling in psoriasis |
|  | PPARG negative regulators and targets |
|  | Model of PPARG related pathways in psoriasis (short version) |
|  | dsRNA induces innate immunity and INFs in alveolar cells |
|  | 1\_Formation of cysts in polycystic kidney disease |
|  | 3\_1\_Beta-cell proliferation and compensation: proliferation |
|  | 4\_1\_Insulin resistance: insulin signaling |
|  | 4\_3\_Insulin resistance: FFA-related insulin resistance |
|  | 2\_Neuron and Schwann cell death |
|  | Model of PPARG signaling in psoriais (tested) |
|  | DIPG overview\_1 (histones) |
|  | DIPG histone 3 mutations |
|  | New Pathway (1) |

---

|  |  |
| --- | --- |
| MedScan ID | 12108602 |
|  | 12471109 |

---

|  |  |
| --- | --- |
| Alias | AMP-dependent protein kinase |
|  | acetyl-CoA carboxylase kinases |
|  | AMP-activated protein kinase |
|  | 2.7.11.27 |
|  | adenosine phosphate activated protein kinase |
|  | AMP-activated acetyl-CoA carboxylase kinase |
|  | hydroxymethylglutaryl coenzyme A reductase (NADPH) kinase |
|  | AMP-regulated protein kinases |
|  | adenosine monophosphate-activated protein kinase |
|  | 2.7.1.111 |
|  | cAMP-independent acetyl-CoA carboxylase kinases |
|  | protein kinase, AMP-activated |
|  | 3-hydroxy-3-methylglutaryl-CoA reductase kinases |
|  | 2.7.1.109 |
|  | protein kinase (adenosine phosphate-activated) |
|  | I-peptide kinase |
|  | (hydroxymethylglutaryl-CoA reductase (NADPH)) kinase |
|  | HMG CoA reductase kinase |
|  | AMPK family kinases |
|  | 2.7.11.31 |
|  | reductase kinases |
|  | reductase kinase |
|  | 3-hydroxy-3-methylglutaryl-CoA reductase kinase |
|  | acetyl CoA carboxylase kinase 3 |
|  | 3-hydroxy-3-methylglutaryl-coenzyme A reductase kinase |
|  | protein kinases (adenosine phosphate-activated) |
|  | [hydroxymethylglutaryl-CoA reductase (NADPH)] kinase |
|  | AMP-dependent protein kinases |
|  | acetyl-coenzyme A carboxylase kinase |
|  | AMPK |
|  | AMPK family kinase |
|  | AMP-activated protein kinases |
|  | 5'-AMP-activated protein kinases |
|  | AMP-regulated protein kinase |
|  | hydroxymethylglutaryl-CoA reductase kinase |
|  | acetyl-CoA carboxylase kinase |
|  | hydroxymethylglutaryl coenzyme A reductase kinase |
|  | AMPKs |
|  | 5'-AMP-activated protein kinase |
|  | 2.7.1.128 |
|  | protein kinases, AMP-activated |
|  | acetyl-coenzyme A carboxylase bound kinase |
|  | cAMP-independent acetyl-CoA carboxylase kinase |
|  | cAMP-independent acetyl-coenzyme A carboxylase kinases |
|  | acetyl-co-enzyme A carboxylase kinase (cAMP-independent) |
|  | AMP-activated acetyl-coenzyme A carboxylase kinase |
|  | ATP:[acetyl-co-enzyme A carboxylase] phospho-transferase |
|  | acetyl-co-enzyme A carboxylase kinase |
|  | acetyl-coenzyme A carboxylase kinases (phosphorylating) |
|  | acetyl-co-enzyme A carboxylase bound kinase |
|  | AMP-activated acetyl-CoA carboxylase kinases |
|  | ATP:[acetyl-co-enzyme A carboxylase] phosphotransferase |
|  | 5-prime-AMP-activated protein kinase |
|  | acetyl-coenzyme A carboxylase kinases |
|  | 5-prime-AMP-activated protein kinases |
|  | cAMP-independent acetyl-coenzyme A carboxylase kinase |
|  | acetyl-coenzyme A carboxylase kinase (phosphorylating) |
|  | acetyl-co-enzyme A carboxylase kinases |
|  | acetyl-CoA carboxylase bound kinase |
|  | AMP-activated acetyl-co-enzyme A carboxylase kinase |
|  | protein kinase,adenosine phosphate activated |
|  | ATP:[acetyl-coenzyme A carboxylase] phosphotransferase |
|  | acetyl-coenzyme A carboxylase bound kinases |
|  | AMP-activated acetyl-co-enzyme A carboxylase kinases |
|  | acetyl-CoA carboxylase kinase (cAMP-independent) |
|  | acetyl-CoA carboxylase kinases (cAMP-independent) |
|  | acetyl-co-enzyme A carboxylase bound kinases |
|  | ATP:[acetyl-CoA carboxylase] phospho-transferase |
|  | ATP:[acetyl-CoA carboxylase] phosphotransferase |
|  | acetyl-coenzyme A carboxylase kinase (cAMP-independent) |
|  | acetyl-co-enzyme A carboxylase kinases (cAMP-independent) |
|  | ATP:[acetyl-coenzyme A carboxylase] phospho-transferase |
|  | AMP-activated acetyl-coenzyme A carboxylase kinases |
|  | I-peptide kinases |
|  | cAMP-independent acetyl-co-enzyme A carboxylase kinases |
|  | acetyl-coenzyme A carboxylase kinases (cAMP-independent) |
|  | cAMP-independent acetyl-co-enzyme A carboxylase kinase |
|  | acetyl-CoA carboxylase bound kinases |

---

|  |  |
| --- | --- |
| EC Number | 2.7.11.31 |
|  | 2.7.11.27 |
|  | 2.7.1.128 |

---

|  |  |
| --- | --- |
| GO ID | 0008602 |
|  | 0050405 |

---

|  |  |
| --- | --- |
| Cell Localization | Cytoplasm |

---
